# Advancing Adsorption and Separation with Modified SBA-15: A Comprehensive Review and Future Perspectives

**DOI:** 10.3390/molecules29153543

**Published:** 2024-07-27

**Authors:** Binjun Liang, Pingxin Zhu, Jihan Gu, Weiquan Yuan, Bin Xiao, Haixiang Hu, Mingjun Rao

**Affiliations:** 1 Ganzhou Key Laboratory of Mine Geological Disaster Prevention and Control and Ecological Restoration, School of Resources and Civil Engineering, Gannan University of Science and Technology, Ganzhou 341000, China; liangbinjun1205@163.com (B.L.); 13755740765@163.com (P.Z.); gujihan@gnust.edu.cn (J.G.); ywqsdut@163.com (W.Y.); hx.hu@gnust.edu.cn (H.H.); 2 Chongyi Green Metallurgy New Energy Co., Ltd., Ganzhou 341300, China; 3 School of Minerals Processing & Bioengineering, Central South University, Changsha 410083, China

**Keywords:** mesoporous silica, SBA-15, modification, adsorption, separation

## Abstract

Mesoporous silica SBA-15 has emerged as a promising adsorbent and separation material due to its unique structural and physicochemical properties. To further enhance its performance, various surface modification strategies, including metal oxide and noble metal incorporation for improved catalytic activity and stability, organic functionalization with amino and thiol groups for enhanced adsorption capacity and selectivity, and inorganic–organic composite modification for synergistic effects, have been extensively explored. This review provides a comprehensive overview of the recent advances in the surface modification of SBA-15 for adsorption and separation applications. The synthesis methods, structural properties, and advantages of SBA-15 are discussed, followed by a detailed analysis of the different modification strategies and their structure–performance relationships. The adsorption and separation performance of functionalized SBA-15 materials in the removal of organic pollutants, heavy metal ions, gases, and biomolecules, as well as in chromatographic and solid–liquid separation, is critically evaluated. Despite the significant progress, challenges and opportunities for future research are identified, including the development of low-cost and sustainable synthesis routes, rational design of SBA-15-based materials with tailored properties, and integration into practical applications. This review aims to guide future research efforts in developing advanced SBA-15-based materials for sustainable environmental and industrial applications, with an emphasis on green and scalable modification strategies.

## 1. Introduction

Mesoporous silica materials, such as MCM-41 (Mobil Composition of Matter No. 41), MCM-48 (Mobil Composition of Matter No. 48), SBA-15 (Santa Barbara Amorphous-15), SBA-16 (Santa Barbara Amorphous-16), etc., as an important class of nanostructured materials, have received widespread attention [1]. These materials have found extensive applications in various fields, including catalysis, drug delivery, membrane separation, biosensing, and adsorption [2]. Mesoporous silica materials have attracted significant attention due to their unique properties, such as high surface area, large pore volume, tunable pore size, and easy surface functionalization [3,4,5]. These properties make them promising candidates for various applications, including catalysis, drug delivery, adsorption, and separation [6,7]. Compared to other porous materials, mesoporous silica offers several advantages. For example, the pore size of mesoporous silica can be easily tailored to accommodate a wide range of guest molecules, from small gas molecules to large biomolecules [3,5]. Moreover, the surface of mesoporous silica can be readily modified with various functional groups, allowing for the fine-tuning of their adsorption and catalytic properties [3,4,7].

In recent years, researchers have begun to pay attention to nanoporous materials with large specific surface areas, such as metal–organic frameworks (MOFs) [8] and mesoporous silica [9]. MOF materials are favored in the adsorption field due to their ultra-high specific surface area and designability. The high surface area provides abundant adsorption sites, while the designability allows for the incorporation of specific functional groups that can enhance the adsorption selectivity and capacity [10,11,12]. Zeolites, another class of nanoporous materials, possess well-defined pore structures and high thermal and chemical stability [13]. They have been widely used in catalysis, gas separation, and ion exchange applications [14]. Activated carbon, known for its high porosity and large surface area, has been extensively employed in water treatment and air purification [15,16]. Mesoporous carbon materials, such as CMK-3 (Carbon Mesostructured by KAIST-3) and CMK-5 (Carbon Mesostructured by KAIST-5), have also gained attention due to their uniform pore structure, high surface area, and good electrical conductivity [17,18]. These properties make them promising candidates for energy storage and conversion applications [17]. Although the aforementioned nanoporous materials have their unique advantages and applications, mesoporous silica stands out due to its distinct features. Compared with MOF materials, the advantage of mesoporous silica lies in its easy surface modification and good tolerance under acidic conditions [19,20]. These properties make mesoporous silica a versatile platform for the development of advanced adsorption and separation materials.

**Figure 1:**
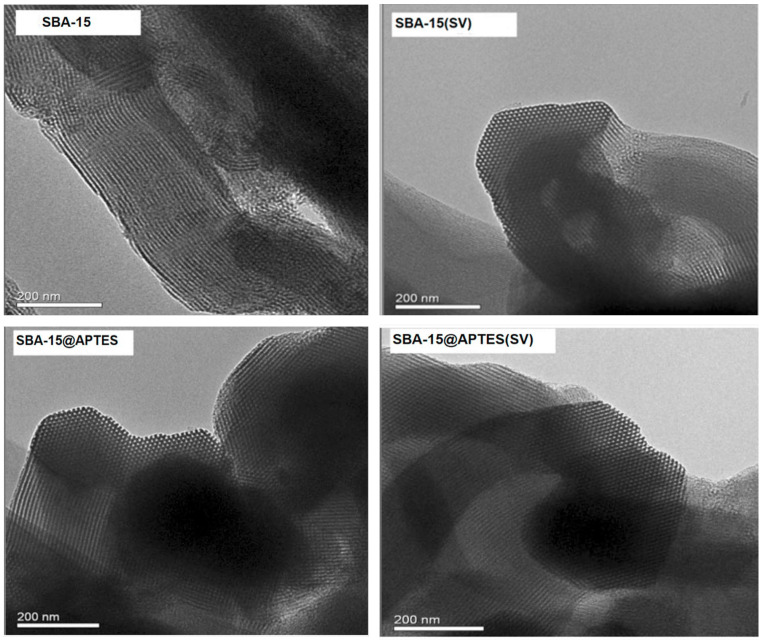
TEM images of SBA-15 and modified SBA-15 [23].

Among many mesoporous silica materials, SBA-15 has received much attention due to its unique structural features and excellent adsorption performance [19,21]. SBA-15 is a mesoporous material with two-dimensional hexagonal arrays of cylindrical pores interconnected with each other by cavities of sizes much smaller than those of their cylindrical pores, first synthesized by Zhao et al. in 1998 [22]. Figure 1 presents the TEM (Transmission Electron Microscopy) images of SBA-15, SBA-15@APTES (SBA-15 modified with 3-Aminopropyltriethoxysilane), SBA-15(SV) (SBA-15 modified with Sodium valproate), and SBA-15@APTES(SV) (SBA-15 modified with both APTES and SV) samples, clearly showing the highly ordered hexagonal pore structure in all materials [23]. These structural features make SBA-15 exhibit unique advantages in the field of adsorption and separation.

In addition to its inherent structural advantages, SBA-15 can be further modified to enhance its adsorption and catalytic properties. The TEM results shown in Figure 1 suggest that the surface functionalization of SBA-15 with APTES and the incorporation of SV did not affect the hexagonal pore structure, consistent with XRD (X-ray Diffraction characterizations) [23]. The modified SBA-15 materials maintain the well-ordered mesoporous structure, indicating successful functionalization without destroying the original framework.

However, SBA-15 still has certain limitations in the adsorption of some specific substances. For example, when using SBA-15 to adsorb heavy metal ions, it mainly relies on physical adsorption, and the adsorption capacity is low [24]. This is because its surface lacks sufficient coordination sites, and the affinity between silanol groups and many heavy metal ions is weak.

Furthermore, SBA-15 also has certain deficiencies in the adsorption of organic pollutants, gas molecules, etc., which affects its actual application effect [20]. Therefore, surface modification of SBA-15 to introduce specific functional groups and improve adsorption performance through chemical interactions with adsorbate molecules has become an important way to expand its application range [24]. It is of great significance to modify the surface of SBA-15 to improve its adsorption performance and broaden its application fields.

Previous reviews have discussed the challenges and progress in the synthesis, characterization, and applications of modified SBA-15 materials for heterogeneous catalysis and adsorption. Mozaffar et al. [25] focused on the synthesis, characterization, and applications of plugged SBA-15 materials for heterogeneous catalysis, including metal and acid modifications. Yuan et al. [26] reviewed recent advances in SBA-15-based composites as heterogeneous catalysts for water decontamination, covering the incorporation of transition metals, metal oxides, and other active components into SBA-15. Moritz et al. [27] investigated the effect of SBA-15 surface modification on the adsorption of 18β-glycyrrhetinic acid, highlighting recent advancements in organic functionalization of SBA-15 for adsorption applications.

Building upon these previous works, this article focuses on the latest progress in the modification of SBA-15 specifically for adsorption applications. Through a comprehensive review and analysis of relevant literature, this article systematically summarizes the recent research on SBA-15 modification from three perspectives: inorganic material modification, organic material modification, and inorganic–organic composite modification. The application of modified SBA-15 in the field of adsorption and separation is discussed in detail, providing a deeper understanding of the structure-property relationships and adsorption mechanisms.

Furthermore, this article offers an outlook on the future research directions of SBA-15 modification. The aim is to guide the development of novel SBA-15-based adsorbents with enhanced performance and expanded application scope. By bridging the gap between fundamental research and practical applications, this review aims to serve as a valuable reference for researchers and engineers working on the development of advanced adsorption materials based on modified SBA-15.

## 2. Structure and Properties of SBA-15

SBA-15 is a highly ordered mesoporous silica material with a hexagonal array of uniform mesopores. Its unique structural and physicochemical properties make it an attractive candidate for various adsorption and separation applications. In this section, we will discuss the synthesis methods, physicochemical properties, and advantages of SBA-15 in adsorption and separation processes.

### 2.1. Synthesis Methods of SBA-15

**Figure 2:**
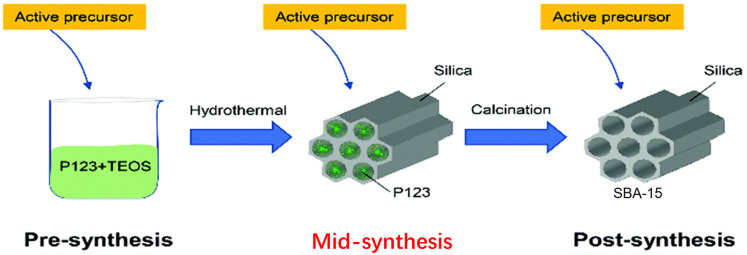
Synthesis route for preparation of SBA-15. Adapted with permission from Ref. [30] under a Creative Commons Attribution—NonCommercial 3.0 Unported License. Copyright (2021) Royal Society of Chemistry.

SBA-15 is typically synthesized using a soft-templating approach, which involves the cooperative self-assembly of amphiphilic triblock copolymers, such as Pluronic P123 (P123), as structure-directing agents (SDAs) and a silica source, like tetraethyl orthosilicate (TEOS), under acidic conditions [28,29]. The synthesis process can be divided into three main stages: pre-synthesis, mid-synthesis, and post-synthesis. In the pre-synthesis stage, P123 and TEOS are combined in an acidic solution, forming a micellar compound. The mixture then undergoes hydrothermal treatment during the mid-synthesis stage, leading to the formation of a hexagonal mesoporous structure with the SDA (P123) incorporated into the silica framework. Finally, in the post-synthesis stage, the template (P123) is removed by calcination at high temperatures, yielding the mesoporous SBA-15 product with the desired hexagonal pore structure (Figure 2).

Various synthesis parameters, such as the type and concentration of the SDA, pH, temperature, and aging time, can be tuned to control the pore size, wall thickness, and morphology of SBA-15 [31]. Additionally, post-synthesis functionalization methods, like grafting or co-condensation, can be employed to introduce various functional groups onto the SBA-15 surface, further enhancing its adsorption and separation capabilities [28,32,33]. By carefully controlling the synthesis parameters and employing appropriate post-synthesis modifications, SBA-15 can be tailored to exhibit optimal structural and functional properties for specific adsorption and separation applications.

### 2.2. Structural and Textural Properties of SBA-15

SBA-15 possesses several unique structural and textural properties that make it an excellent adsorbent and separation material. It exhibits a highly ordered hexagonal mesoporous structure with uniform pore channels and an interconnected pore network [34,35]. This well-defined pore structure, combined with its high specific surface area (typically 500–1000 m^2^/g) and large pore volume (0.6–1.2 cm^3^/g), facilitates efficient diffusion and transport of adsorbates, leading to enhanced adsorption kinetics and capacity [35,36]. The thick pore walls (typically 3–6 nm) and high thermal and hydrothermal stability of SBA-15 enable the size-selective adsorption and separation of large molecules in harsh environments [37,38]. Furthermore, the surface of SBA-15 is rich in silanol groups, which serve as reactive sites for surface functionalization and modification [39]. By grafting various functional groups or incorporating active species, the surface chemistry of SBA-15 can be tailored to achieve enhanced adsorption selectivity, specificity, and capacity [39,40]. The uniform mesoporous structure also provides abundant sites for adsorption and surface functionalization [41].

Table 1 summarizes the key structural and textural properties of SBA-15 in comparison with other representative mesoporous silica materials, such as MCM-41, MCM-48, MCF, KIT-6, and HMS. SBA-15 stands out with its larger tunable pore size range, thicker pore walls, and highly ordered hexagonal pore structure, which collectively contribute to its superior thermal stability, mechanical strength, and adsorption performance.

The larger pore sizes of SBA-15 allow for the adsorption and separation of larger molecules that might not fit in materials with smaller pores, such as MCM-41, MCM-48, and HMS. This wide range of tunable pore sizes also provides flexibility in tailoring the material for specific applications. The thicker pore walls contribute to its enhanced thermal stability and durability, making it suitable for use in more demanding conditions compared to materials with thinner walls.

In summary, the key advantages of SBA-15 over other mesoporous silica materials, as shown in Table 1, include its larger tunable pore size range, thicker pore walls, highly ordered hexagonal pore structure, and abundant surface silanol groups [42,43]. These structural and textural properties make SBA-15 a promising material for various adsorption and separation applications, which will be discussed in detail in the following sections. Compared to other materials like MCM-41, MCM-48, MCF, KIT-6, and HMS, SBA-15 stands out with its unique combination of textural features that contribute to its superior thermal stability, mechanical strength, and adsorption performance

### 2.3. Advantages of SBA-15 in Adsorption and Separation

The unique structural and physicochemical properties of SBA-15 offer several advantages in adsorption and separation processes:

a. High adsorption capacity and selectivity: Size-selective adsorption and efficient removal of pollutants

The large surface area and pore volume of SBA-15 provide ample adsorption sites, while the uniform and tunable pore size allows for size-selective adsorption and separation of target molecules [40,41]. This is particularly important for the efficient removal of specific pollutants from complex mixtures.

b. Fast adsorption kinetics: Rapid diffusion and transport of adsorbates

The interconnected pore structure and large pore size of SBA-15 facilitate rapid diffusion and transport of adsorbates, leading to fast adsorption kinetics [49]. This is crucial for practical applications where high throughput and short residence times are required.

c. Versatile surface chemistry: Tailoring adsorption properties through functionalization

The abundant surface silanol groups on SBA-15 enable a wide range of surface functionalization and modification strategies, allowing for the tailoring of adsorption properties for specific applications [50,51]. This versatility greatly expands the potential applications of SBA-15 in diverse adsorption and separation scenarios.

d. Excellent stability and reusability: Long-term use and cost-effectiveness

The thick pore walls and high hydrothermal stability of SBA-15 ensure its structural integrity during adsorption and regeneration cycles, enabling long-term use and reusability [52]. This is essential for the development of cost-effective and sustainable adsorption and separation processes.

e. Compatibility with various modification methods: Rational design of multi-functional adsorbents

SBA-15 can be easily modified with inorganic, organic, and inorganic–organic composite species, expanding its potential applications in adsorption and separation processes [53]. This compatibility allows for the rational design and synthesis of multi-functional adsorbents and separation materials based on SBA-15.

In summary, the exceptional structural and physicochemical properties of SBA-15, including its high surface area, tunable pore size, thick pore walls, and versatile surface chemistry, make it a highly promising material for a wide range of adsorption and separation applications. These unique features enable SBA-15 to exhibit superior adsorption capacity, selectivity, kinetics, stability, and multi-functionality, paving the way for the development of advanced and efficient adsorption and separation processes.

## 3. Surface Modification Strategies of SBA-15

The surface modification of SBA-15 is a crucial approach to tailor its properties and expand its applications in various fields. The modification strategies can be broadly classified into three categories: inorganic modification [54], organic modification [55], and inorganic–organic composite modification [56].

### 3.1. Inorganic Modification

The inorganic modification of SBA-15 aims to enhance its structural stability, adsorption capacity, and catalytic performance by introducing inorganic species into the framework. This category includes metal oxide and non-metal element modification.

#### 3.1.1. Metal Oxide and Non-Metal Element Modification

Metal oxide, metal nanoparticle, and non-metal element modification of SBA-15 can be achieved through various methods, including direct synthesis [54], post-synthesis grafting [57], doping [58], and impregnation [59]. These modifications aim to enhance the structural stability, adsorption capacity, and catalytic performance of SBA-15. Table 2 summarizes the research on the modification methods and performance of several representative metal oxides and non-metal elements on SBA-15, providing a clear overview of the diverse applications of these modified materials.

The incorporation of metal oxides and non-metal elements into SBA-15 has led to significant improvements in catalytic performance and adsorption capacity across a wide range of applications. The following examples showcase the diversity and potential of these modified SBA-15 materials.

Yadav et al. [60] used a one-step wet impregnation method to prepare alumina-loaded SBA-15 molecular sieves. The catalytic activities of these metal oxide-loaded SBA-15 catalysts were evaluated under the same conditions for the alkylation reaction. The results showed that all three catalysts exhibited excellent conversion and selectivity to form monoalkylated products, with the highest conversion achieved within 1 min. Notably, the catalytic activity remained unchanged after repeated use, indicating good stability and reusability.

Yao et al. [61] prepared a series of SBA-15-supported mesoporous catalysts by ultrasonic impregnation for the degradation of polyethylene terephthalate (PET) to produce bis(2-hydroxyethyl) terephthalate (BHET). The ultrasonic impregnation method ensured a uniform dispersion of the active species on the SBA-15 support, leading to enhanced catalytic performance. The experimental results showed that the 5%ZnO/SBA-15 catalyst exhibited the best stability and maintained high catalytic activity during the recycling process, demonstrating its potential for practical applications in plastic waste management.

**Figure 3:**
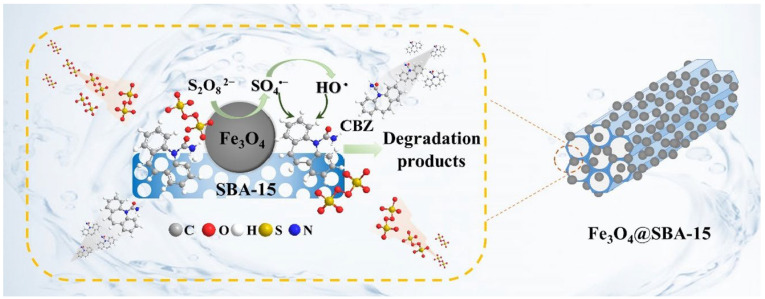
A core–shell-like nanocomposite comprised of Fe_3_O_4_ and SBA-15 [62].

Huang et al. [62] prepared an Fe_3_O_4_-wrapped mesoporous molecular sieve catalyst (Fe_3_O_4_@SBA-15) using SBA-15 as a carrier for the activation of persulfate (PS). The synthesis effect diagram of Fe_3_O_4_@SBA-15 is shown in Figure 3, illustrating its core–shell-like nanocomposite structure.

The mesoporous structure of SBA-15 provided a high surface area and abundant pore channels for the uniform distribution of Fe_3_O_4_ nanoparticles, while the Fe_3_O_4_ core enhanced the magnetic properties and catalytic activity of the composite. Under optimized conditions (Fe_3_O_4_/SBA-15 mass ratio of 3:1, pH 3.0, temperature 25 °C, initial PS mass concentration 300 mg/L, and catalyst mass concentration 0.50 g/L), Fe_3_O_4_@SBA-15 achieved a remarkable removal rate of carbamazepine (100%) within 30 min, owing to the efficient activation of PS and the synergistic effect between Fe_3_O_4_ and SBA-15. Moreover, the catalyst maintained its high activity during six consecutive cycles, demonstrating its excellent stability and reusability for water treatment applications.

**Figure 4:**
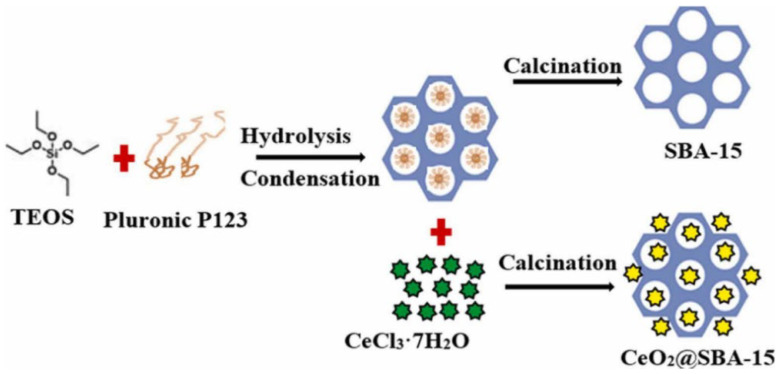
Experimental procedure of synthesis of SBA-15 and CeO_2_@SBA-15 [64].

Several other metal oxides have been successfully incorporated into SBA-15 for various applications. Bepari et al. [67] prepared Co_3_O_4_/SBA-15 catalysts using the nicotinic acid metal salt method, which significantly improved the catalytic activity and selectivity for the epoxidation of styrene, with further enhancement by Au deposition due to the Au-Co synergistic effect. Tsai et al. [68] studied the properties of metal oxide-impregnated SBA-15 (Fe, Co, Ni) and their performance in isopropanol decomposition, observing increased pore wall thickness and decreased pore diameter compared to pure SBA-15. Titiya et al. [69] synthesized Fe-γ-CS-SBA-15 by the hydrothermal method for the efficient removal of methylene blue dye, achieving a 96% removal rate under optimal conditions. Lemoupi et al. [64] synthesized CeO_2_@SBA-15 catalyst for the transesterification of palm fatty oil (PFO) with methyl esters to prepare biodiesel, achieving a maximum yield of 80.2% under optimized conditions (Figure 4). This study highlights the potential of CeO_2_@SBA-15 as an efficient and eco-friendly catalyst for the production of biodiesel from renewable resources, contributing to the development of sustainable energy solutions.

In addition to metal oxides, non-metal elements such as phosphorus have also been explored as modifiers for SBA-15. Wang et al. [70] synthesized SBA-15 using the silicon element from fly ash as the silicon source and surface-modified it with phosphonoacetic acid (PAA) to obtain the adsorption material PAA-SBA-15. The results showed that the presence of Fe(III) had the strongest interference on the adsorption of rare earth ions by PAA-SBA-15, while the adsorption–desorption cycle experiment demonstrated good reusability of the adsorbent.

**Figure 5:**
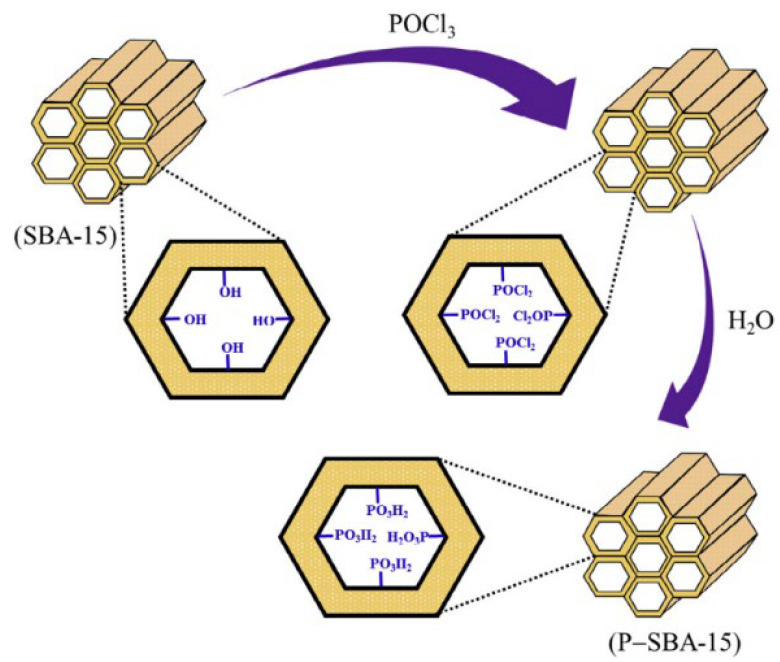
Scheme of preparation of phosphorous acid modified mesoporous SBA-15 (P-SBA-15) [66].

Gao et al. [66] synthesized inorganic hypophosphorous acid modified mesoporous SBA-15 (P-SBA-15) by a simple and economical post-grafting method and studied its adsorption behavior for rare earth ion Gd(III). P-SBA-15 exhibited excellent performance in terms of adsorption capacity and kinetics for Gd(III), reaching adsorption equilibrium within 2 min. The reusability test showed that this mesoporous adsorbent had good reusability. The preparation scheme of P-SBA-15 is shown in Figure 5.

These studies demonstrate the significant impact of metal oxide and non-metal element modification on the performance of SBA-15 in various catalytic and adsorption applications. By carefully selecting the appropriate modification method and optimizing the synthesis conditions, researchers can develop highly efficient and stable SBA-15-based materials for a wide range of industrial and environmental applications. As research in this field continues to progress, it is expected that new and innovative modifications will be developed, further expanding the potential applications of SBA-15-based materials. However, challenges such as cost-effectiveness, scalability, and long-term stability under practical conditions still need to be addressed to facilitate the widespread implementation of these advanced materials.

#### 3.1.2. Noble Metal Nanoparticle Modification

The incorporation of noble metal nanoparticles, such as Pd and Pt, into SBA-15 has been explored to enhance its catalytic performance in various reactions. The synergistic effect between these nanoparticles and the well-defined porous structure of SBA-15 results in improved catalytic activity, selectivity, and stability.

For example, Zhang et al. [71] prepared Pd and Pt nanoparticles supported on mesoporous silica molecular sieve SBA-15 and applied them to the catalytic reduction of bromate. The activity studies showed that the Pt/SBA-15 catalyst had the highest catalytic activity among the tested catalysts, and it maintained its activity with only a slight loss of 7.8% after five repeated trials, indicating its reusability in the catalytic reduction of bromate in aqueous solutions. Similarly, Kuppusamy et al. [72] prepared a series of Pt-Ni-loaded Ti-SBA-15 catalysts with different Si/Ti ratios and studied their performance in the dry reforming of methane. Pt-Ni/Ti-SBA-15 showed higher catalytic activity and stability compared with other catalysts, demonstrating the beneficial effect of titanium doping on the catalytic activity, stability, and anti-coking performance of SBA-15.

In another study, Chen et al. [73] synthesized phosphotungstic acid modified short mesoporous HPW/Zr-SBA-15 by one-step method and used it to catalyze the synthesis of bisphenol F from formaldehyde and phenol. Under the conditions of reaction time of 120 min, phenol/formaldehyde molar ratio of 15:1, catalyst/formaldehyde mass ratio of 1:2, and reaction temperature of 90 °C, using 30% HPW/Zr-SBA-15 to catalyze the synthesis of bisphenol F from phenol and formaldehyde, the yield of bisphenol F was 98.36%, and the yield still reached 87.36% after four times of repeated use of the catalyst, indicating good reusability. It is a highly efficient catalyst for the synthesis of bisphenol F from formaldehyde and phenol in the direction of green chemical industry development.

### 3.2. Organic Modification

In addition to inorganic modification, the organic modification of SBA-15 is another effective approach to enhance its adsorption capacity, selectivity, and compatibility with target molecules. The three main approaches for organic modification are the introduction of organic functional groups, organic small molecule modification, and polymer modification.

#### 3.2.1. Incorporation of Organic Functional Groups

The introduction of organic functional groups onto the SBA-15 surface can be achieved through post-synthesis grafting or co-condensation methods. Common organic functional groups include amino, thiol, carboxyl, and phenyl groups. The incorporation of these functional groups can enhance the adsorption capacity and selectivity of SBA-15 towards specific target molecules through various interactions, such as electrostatic interactions, hydrogen bonding, and π-π stacking.

For example, Chen et al. [74] successfully prepared amino-modified SBA-15 mesoporous molecular sieve with a pore size of 7.85 nm and a specific surface area of 574.75 m^2^/g. Using the amino-modified SBA-15 mesoporous molecular sieve as the carrier for immobilized enzymes, the optimal conditions for the immobilization of lysozyme and lipase were determined, respectively. The co-immobilized dual enzymes were added to the epoxy resin to prepare an epoxy composite coating. The test showed that adding the co-immobilized dual enzymes on amino-modified SBA-15 mesoporous molecular sieve to the epoxy resin could improve its corrosion resistance.

**Figure 6:**
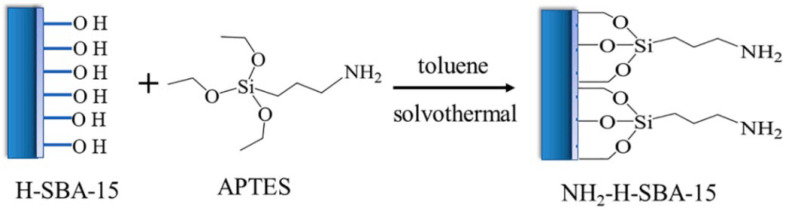
Synthesis process of the NH_2_-H-SBA-15 [75].

Similarly, Wang et al. [75] prepared an NH_2_-functionalized hydroxylated mesoporous SBA-15 (NH_2_-H-SBA-15) adsorbent by post-grafting method, in which aminosilane reacted with the silanol groups on the surface of hydroxylated SBA-15. The synthesis process of NH_2_-H-SBA-15 is shown in Figure 6. The adsorbent was applied in the field of uranium adsorption and exhibited good selectivity and competitive adsorption capacity in both artificial and natural seawater. NH_2_-H-SBA-15 demonstrated good adsorption performance over a wide pH range and excellent adsorption reusability, highlighting the potential of amino-functionalized SBA-15 in the selective removal of uranium from complex aqueous systems.

In another study, Safora et al. [76] synthesized a melamine functionalized mesoporous silica-SBA-15 adsorbent (Melamine-MS-SBA-15). MS-SBA-15 was synthesized by hydrothermal method and functionalized with SBA-15-Melamine under toluene reflux conditions for the removal of Cr(VI) from wastewater. The experimental results showed that after the surface of MS-SBA-15 was functionalized with melamine, both the adsorption efficiency and adsorption capacity were improved. The maximum adsorption capacity for Cr(VI) was about 50 mg/g. SBA-15 performed well for Cr(VI) removal after melamine functionalization, which was due to the transformation from physical adsorption to chemical adsorption.

These studies demonstrate the significant impact of organic functional group modification on the adsorption performance of SBA-15. By carefully selecting the appropriate functional groups and optimizing the modification conditions, researchers can develop highly efficient and selective SBA-15-based adsorbents for a wide range of target pollutants in various environmental applications.

#### 3.2.2. Modification with Organic Small Molecules

Organic small molecule modification involves the immobilization of small organic molecules, such as cyclodextrins [77], calixarenes [78], and crown ethers [79], onto the SBA-15 surface. These organic molecules possess unique host–guest recognition properties, which can significantly improve the adsorption selectivity of SBA-15 towards specific guest molecules. For example, cyclodextrin-modified SBA-15 has been reported to exhibit enhanced adsorption capacity and selectivity towards organic pollutants, such as phenols and dyes, through the formation of inclusion complexes [77].

**Figure 7:**
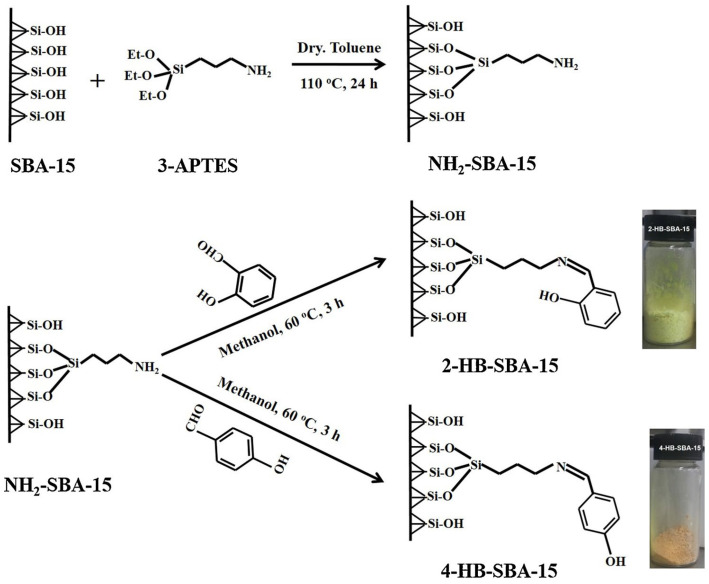
Preparation of NH2-SBA-15, 2-HB-SBA-15, and 4-HB-SBA-15 [80].

Parambadath et al. [80] investigated the selective adsorption of eight heavy metal ions by SBA-15 modified with 2-hydroxybenzaldehyde (2-HB) and 4-hydroxybenzaldehyde (4-HB). As shown in Figure 7, these molecules were immobilized onto amino-functionalized SBA-15 (NH_2_-SBA-15) through a Schiff base condensation reaction, yielding 2-HB-SBA-15 and 4-HB-SBA-15. The difference in the position of the hydroxyl group in 2-HB and 4-HB leads to distinct chelation possibilities with metal ions. Competitive adsorption experiments were carried out using 2-HB-SBA-15 and 4-HB-SBA-15 from a mixture containing Mn(II), Co(II), Ni(II), Cu(II), Cr(III), Zn(II), Pb(II), and Cd(II) ions under pH 2–6 conditions. The results showed that the two materials exhibited different affinities towards metal ions under various pH conditions. The 2-HB-SBA-15 exhibited 100% selectivity towards Pb(II) ions at pH 2, while the selectivity turned to Cu(II) at higher pH. In contrast, 4-HB-SBA-15 showed an affinity towards Cr(III) and Pb(II) at pH 2, while a distributed selectivity was observed with major portions to Cr(III), Pb(II), and Cu(II) at higher pH conditions.

The difference in molar adsorption selectivities of 2-HB-SBA-15 and 4-HB-SBA-15 clearly indicates the crucial role of the chelation effect in the selective adsorption of metal ions under identical conditions. This study highlights the advantages and potential of organic small molecule modification in tuning the selective adsorption performance of SBA-15 by rational design of the modifying molecules [80]. This work further demonstrates the potential of organic small molecule modification in improving the selective adsorption performance of SBA-15, complementing the previous studies on cyclodextrin, calixarene, and crown ether modified SBA-15 materials.

#### 3.2.3. Polymer Grafting and In Situ Polymerization

Polymer modification involves the grafting of polymers onto the SBA-15 surface or the in situ polymerization of monomers within the SBA-15 pores. The incorporation of polymers can significantly enhance the adsorption capacity, selectivity, and stability of SBA-15 [81,82,83].

For instance, Huang et al. [84] used composite molecular sieve SBA-15/Y as the carrier, polyethyleneimine as the functional monomer, and epichlorohydrin as the cross-linking agent to successfully synthesize a lanthanum ion-imprinted polymer (La-IIP/SBA-15/Y) for the recovery and enrichment of lanthanum ions. The composite molecular sieve SBA-15/Y, as a new material, can integrate the properties of two molecular sieves. The results showed that La-IIP/SBA-15/Y had good selectivity and experimental elution regeneration performance.

**Figure 8:**
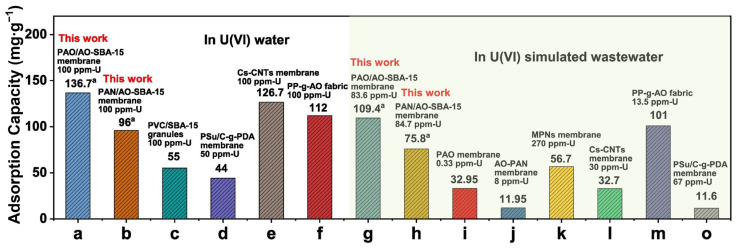
Comparative studies of adsorption on PAN/AO-SBA-15 and PAO/AO-SBA-15 membranes with other reported adsorbents in U (VI) water or simulated wastewater [81]. a: Adsorption capacity is calculated by q_m_·S/m, where q_m_ is the maximum adsorption capacity (mg/m^2^), S is the membrane area (m^2^), and m is the mass (g) of the membrane before adsorption.

Wang et al. [81] prepared a precursor membrane adsorbent (PAN/AO-SBA-15) using polyacrylonitrile (PAN) as a binder and amidoxime (AO) functionalized SBA-15 as a powdered adsorbent by phase transformation method. The PAN/AO-SBA-15 membrane was further modified to prepare a PAO (Poly amidoxime)/AO-SBA-15 membrane by the amidoxime method. The experiments showed that PAO/AO-SBA-15 exhibited high adsorption capacity in pure U(VI) aqueous solution, and the PAO/AO-SBA-15 membrane showed better selectivity and more competitive adsorption capacity. Figure 8 presents the comparative studies of adsorption on PAN/AO-SBA-15 and PAO/AO-SBA-15 membranes with other reported adsorbents in U(VI) water or simulated wastewater.

**Figure 9:**
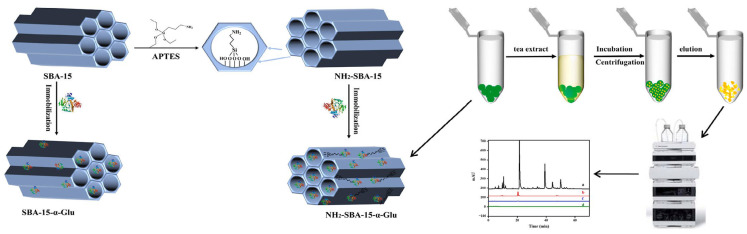
Synthesis and screening process of NH_2_-SBA-15-α-Glu microreactor [85].

In another study, Fan et al. [85] modified SBA-15 with APTES to construct an immobilized enzyme microreactor (NH2-SBA-15-α-Glu) based on the electrostatic adsorption of α-Glu. By modifying SBA-15, α-Glu was dispersed on the surface and inner walls of the two-dimensional channels of NH_2_-SBA-15 by electrostatic adsorption to construct the NH_2_-SBA-15-α-Glu microreactor. Under optimum conditions, the quantity of immobilized α-Glu by NH_2_-SBA-15 was 39.93 μg/mg, which was much higher than unmodified SBA-15 (15.92 μg/mg). In addition, the thermal stability and acid–base resistance of the microreactor were also greatly improved, and it could be reused, retaining 76.1% of the enzyme activity after 7 cycles. The synthesis process is shown in Figure 9.

### 3.3. Inorganic–Organic Composite Modification

While inorganic and organic modifications have been widely explored separately, the combination of both strategies, known as inorganic–organic composite modification, has emerged as a promising approach to develop multifunctional SBA-15-based materials with enhanced adsorption [86], catalytic [87], and sensing properties [88]. This section discusses the preparation methods and synergistic effects of inorganic–organic composite SBA-15 materials.

#### 3.3.1. Preparation of Inorganic–Organic Composite Materials

Inorganic–organic composite SBA-15 materials can be prepared through various methods, such as post-synthesis grafting [89], co-condensation [90], and impregnation [91]. The post-synthesis grafting method involves modifying the surface of SBA-15 with organic functional groups, which can further coordinate with metal species to form composite materials with enhanced catalytic or adsorptive properties.

**Figure 10:**
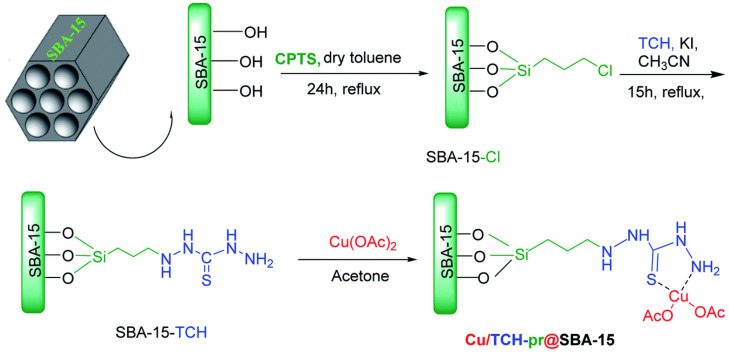
Schematic diagram of the synthesis process of Cu/TCH-pr@SBA-15. Reprinted from Ref. [92] with permission from the Royal Society of Chemistry under a Creative Commons Attribution—NonCommercial 3.0 Unported License, copyright (2021).

A typical example of post-synthesis grafting is the preparation of thiol-functionalized SBA-15 and its metal complexes, as shown in Figure 10 [92]. SBA-15 is first modified with chloropropyl groups by reacting with chloropropyltriethoxysilane (CPTS) under dry toluene reflux for 24 h, resulting in SBA-15-Cl. Subsequently, SBA-15-Cl reacts with TCH and KI under reflux for 15 h to form thiol-functionalized SBA-15 (SBA-15-TCH). The grafted thiol groups can further coordinate with metal species like Cu(OAc)_2_ in acetone to form metal-loaded catalysts such as Cu/TCH-pr@SBA-15. These composite materials combine the advantages of both inorganic and organic components, leading to enhanced catalytic performance.

Many other inorganic–organic composite SBA-15 materials prepared by post-synthesis grafting have also shown excellent performance in various applications. For instance, Pd nanoparticles immobilized on imidazolium-functionalized SBA-15 exhibited a high yield of 95% for the Suzuki carbon–carbon coupling reaction under mild conditions [93]. Phosphonium-modified SBA-15 efficiently catalyzed the Knoevenagel condensation, achieving a 92% yield of the desired product with high selectivity [55]. Pyridinium-sulfonic acid functionalized SBA-15 was developed as a robust solid acid catalyst for esterification, retaining 88% yield even after five reaction cycles [89].

Apart from post-synthesis grafting, other methods like co-condensation and impregnation have also been employed to prepare inorganic–organic composite SBA-15 materials with bifunctional properties. The co-condensation method involves the simultaneous condensation of organosilanes and TEOS in the presence of a structure-directing agent, leading to the direct incorporation of organic functional groups into the silica framework. This one-pot synthesis approach is relatively simple and can achieve high loading of organic groups, but may result in a less ordered mesoporous structure compared to post-synthesis grafting [93,94,95]. On the other hand, the impregnation method involves first preparing metal oxide-modified SBA-15 by techniques like wet impregnation or deposition–precipitation, followed by the grafting of organic moieties. This stepwise approach allows for better control over the composition and structure of the resulting composite materials [92].

Table 3 lists some representative examples of inorganic–organic composite SBA-15 materials prepared by different methods and their applications in catalysis. These composite materials exhibit enhanced performance compared to their individual counterparts, owing to the synergistic effects between the inorganic and organic components.

In summary, the inorganic–organic composite modification of SBA-15 has emerged as a versatile and effective strategy to develop multifunctional materials with tailored properties. By judiciously combining inorganic and organic components through various methods, these composite SBA-15 materials can exhibit improved catalytic, adsorptive, and sensing performance. However, the preparation of such materials often involves complex procedures and may require careful optimization to achieve the desired properties. Future research efforts should focus on the rational design, synthesis, and characterization of these composite materials, as well as elucidating the structure–property relationships and synergistic mechanisms, to fully exploit their potential in various applications.

#### 3.3.2. Synergistic Effects and Applications

The incorporation of both inorganic and organic species into the SBA-15 framework can lead to synergistic effects, enhancing the overall performance of the material in various applications. The following examples demonstrate the synergistic effects of inorganic–organic composite modification on the adsorption, catalytic, and drug delivery properties of SBA-15 [22,26,101].

Liu et al. [102] prepared a magnetic amine-functionalized adsorbent (Fe_3_O_4_@SBA-15-PDA/HBP-NH_2_) by loading Fe_3_O_4_ nanoparticles on hyperbranched polymer (HBP-NH_2_) functionalized mesoporous silica molecular sieve SBA-15. This adsorbent was used for the adsorption of chromium (VI) and uranium (VI). The synergistic effect of combining Fe_3_O_4_ nanoparticles and hyperbranched polymer functionalization led to enhanced adsorption capacities for both U(VI) and Cr(VI). The magnetic Fe_3_O_4_ nanoparticles facilitated the easy separation and recovery of the adsorbent, while the hyperbranched polymer provided abundant amine groups for effective chelation of metal ions. The experimental results showed that the adsorbent had a strong affinity for the two target pollutants, with maximum adsorption capacities of 77.4 mg/g and 66.5 mg/g for U(VI) and Cr(VI), respectively.

**Figure 11:**
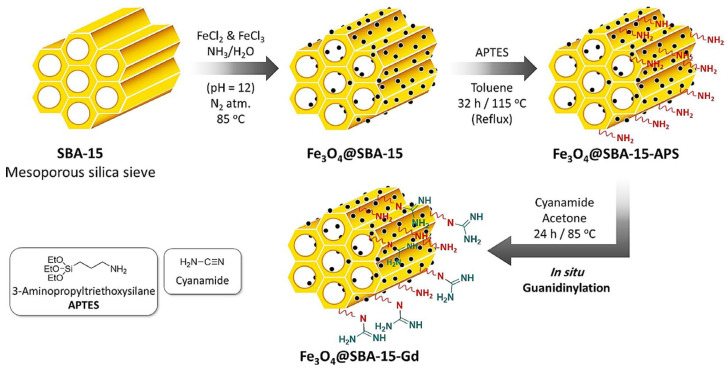
Schematic presentation of successive stages of the preparation route of Fe_3_O_4_@SBA-15-Gd adsorbent system [103].

Similarly, Fereshte et al. [103] synthesized SBA-15 by the hydrothermal method and prepared in situ magnetized Fe_3_O_4_@SBA-15 using Fe_3_O_4_ magnetic nanoparticles (MNP). The synthesized material was then modified with APTES to obtain Fe_3_O_4_@SBA-15-NH_2_. Finally, a magnetic Fe_3_O_4_@SBA-15-Gd nano-adsorbent was prepared via nucleophilic addition. The preparation process is shown in Figure 11. This adsorbent achieved maximum adsorption capacities of 344.82 mg/g and 303.03 mg/g for Cu(II) and Pb(II), respectively, demonstrating the synergistic effect of magnetic Fe_3_O_4_ nanoparticles and gadolinium functionalization on the adsorption performance of SBA-15. Moreover, this magnetic adsorbent exhibited good reusability, highlighting its potential for practical applications in heavy metal removal.

Moreover, the development of inorganic–organic composite SBA-15 materials has shown promising potential in catalytic applications, such as the photocatalytic degradation of organic pollutants and the electrocatalytic reduction of CO_2_ [104,105]. The synergistic combination of semiconductor nanoparticles (e.g., TiO_2_, CdS) and organic sensitizers (e.g., porphyrins, metal–organic frameworks) on SBA-15 has been reported to enhance the photocatalytic activity and stability under visible light irradiation [104,106]. Similarly, the incorporation of metal nanoparticles (e.g., Cu, Ag) and conductive polymers (e.g., polyaniline, polypyrrole) into SBA-15 has been shown to improve the electrocatalytic performance and selectivity for CO_2_ reduction [105,107,108].

**Figure 12:**
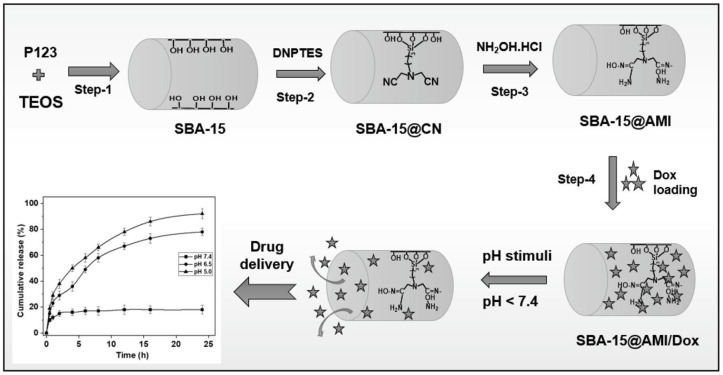
Preparation and modification of SBA-15 materials, respectively [109].

In addition to adsorption and catalytic applications, inorganic–organic composite SBA-15 materials have also been explored for drug delivery. Selvakumari et al. [109] synthesized amidoxime (AMI) functionalized mesoporous silica nanoparticles (SBA-15@AMI NPs) by surface modification method and examined their drug loading and pH-responsive release behavior using doxorubicin (Dox) as a model anti-cancer drug. The preparation and modification process of SBA-15@AMI NPs is illustrated in Figure 12. The synergistic combination of amidoxime functionalization and the mesoporous structure of SBA-15 resulted in a pH-responsive controlled drug delivery system with potential applications in cancer treatment. The biocompatibility and cellular uptake behavior of SBA-15@AMI NPs were studied using the MCF-7 cell line. Overall, the prepared SBA-15@AMI nanoparticles demonstrated their potential as a pH-responsive controlled drug delivery system for cancer treatment.

These studies highlight the versatility and effectiveness of inorganic–organic composite modification in tailoring the properties and performance of SBA-15 for a wide range of applications. By carefully selecting the inorganic and organic components and optimizing the synthesis conditions, researchers can develop multifunctional SBA-15-based materials with enhanced adsorption, catalytic, and drug delivery properties, tailored to specific target applications.

In summary, the inorganic–organic composite modification strategies discussed in this section have demonstrated their versatility and effectiveness in tailoring the properties and performance of SBA-15 for a wide range of applications. By carefully selecting the inorganic and organic components and optimizing the synthesis conditions, researchers can develop multifunctional SBA-15-based materials with enhanced adsorption, catalytic, and drug delivery properties, tailored to specific target applications. The successful application of these composite materials highlights the immense potential of inorganic–organic composite modification strategies in fields such as environmental remediation, drug delivery, and chemical synthesis.

## 4. Limitations and Future Perspectives of SBA-15

SBA-15 and its modified variants have shown remarkable advantages and extensive applications in various fields, such as catalysis, adsorption, and drug delivery [29,110]. However, there are still several limitations that need to be addressed for their widespread industrial implementation. These limitations can be broadly categorized into high cost, poor physical properties, and synthesis and functionalization challenges. In this section, we will discuss these limitations in detail and explore potential strategies to overcome them, with a focus on specific examples and applications.

### 4.1. High Cost and Poor Physical Properties

Despite its numerous advantages, SBA-15 faces several limitations that hinder its widespread industrial implementation. The high production cost, due to expensive structure-directing agents like P123, makes large-scale production economically challenging [25,111]. SBA-15 also exhibits limited thermal and hydrothermal stability compared to zeolites, especially in demanding reactions like biomass conversion [112]. The relatively fragile nature of SBA-15 can lead to degradation by abrasion, particularly in slurry liquid phase reactions [52]. Additionally, SBA-15 generally exhibits lower acidity compared to zeolites, which can limit its effectiveness in certain catalytic applications, such as the adsorption of uranium from aqueous solutions. Pure siliceous SBA-15 has an electronically neutral framework and lacks Brønsted acidity, hindering its catalytic activity [112,113]. The pore structure of SBA-15 can sometimes hinder diffusion, which could be improved by widening intrawall pores. Furthermore, unexpected instability in water, even at room temperature, has been observed despite the thick walls of SBA-15 [25,52].

### 4.2. Synthesis and Functionalization Challenges

Synthesis and functionalization of SBA-15 also present several challenges. There are difficulties in obtaining nanoparticles from the active phase introduced into SBA-15 and keeping them stable in acidic conditions during synthesis [25,113]. The highly acidic conditions required for SBA-15 synthesis (pH < 1) can make it challenging to incorporate certain metal oxides that are strongly affected by pH [33]. For example, the direct-synthesis and pH-adjustment methods have been developed to prepare Zr-SBA-15 materials, as zirconia is sensitive to highly acidic conditions [114]. Moreover, the incorporation of metal oxides or nanoparticles can lead to partial pore blocking, reducing the effective surface area and pore volume [115].

### 4.3. Future Perspectives and Improvement Strategies

To address these limitations and enhance the applicability of SBA-15, several strategies can be pursued. Research should focus on finding alternative, less expensive structure-directing agents or developing synthesis routes that reduce or eliminate the need for costly templates [116]. Enhancing thermal and hydrothermal stability through the incorporation of tri- and tetra-valent heteroatoms (e.g., Al, Ti, Zr) into the silica wall of SBA-15 could improve its stability and expand its application range. For instance, the incorporation of ceria nanoparticles has been shown to improve the thermal stability of SBA-15 [52,113]. Work on shaping techniques for SBA-15 materials could enhance their mechanical stability, making them more suitable for industrial applications [25]. The tailored synthesis of SBA-15 rods using different types of acids could also be explored to address some of the limitations [33].

Further research into metal-substituted SBA-15 materials could lead to more efficient catalytic applications. For example, the transition from Al-SBA-15 to Ga-SBA-15 has been studied to improve the acidity and catalytic performance of SBA-15 [29]. Developing new strategies for nanoparticle incorporation, such as the direct modification (DM) strategy for Pt nanoparticles, could improve the content and dispersion of active phases within SBA-15 channels [25,29]. Exploring synthesis routes at less acidic pH (around 5) could facilitate the incorporation of pH-sensitive metal oxides [29,33]. Finally, developing methods to increase pore diameters and optimize pore structures could address diffusion limitations and improve catalytic performance [25,29].

By addressing these limitations and pursuing the suggested improvement strategies, the applicability and performance of SBA-15 and its modified variants can be significantly enhanced, leading to their wider adoption in various industrial applications, such as catalysis, adsorption, and drug delivery.

## 5. Adsorption and Separation Performance and Applications of SBA-15

SBA-15, with its unique structural and physicochemical properties, has shown remarkable performance in the adsorption and separation of various substances, including organic pollutants, heavy metal ions, gases, and biomolecules. This section discusses the adsorption and separation performance of SBA-15 in different applications and highlights its potential in wastewater treatment, environmental remediation, and other related fields.

### 5.1. Adsorption of Organic Pollutants and Heavy Metal Ions

SBA-15 has been effectively used for the removal of various organic pollutants and heavy metal ions from aqueous solutions. Functionalized SBA-15 materials exhibit high surface areas, large pore volumes, and tunable surface chemistry, resulting in significant adsorption capacities and selectivity for these pollutants [39,116].

#### 5.1.1. Adsorption of Organic Pollutants

SBA-15 has been effectively used for the removal of various organic pollutants, such as phenols, dyes, humic acid, and volatile organic compounds (VOCs), from aqueous solutions [117,118]. For instance, aminopropyl-functionalized SBA-15 has shown high adsorption capacity for phenol and p-nitrophenol from aqueous solutions, owing to the strong interactions between the amino groups and the phenolic compounds [24]. Aminopropyl-functionalized SBA-15 is typically prepared using silane coupling agents such as APTES [119]. The functionalization process can vary depending on synthesis conditions, including temperature, pH, and reagent concentrations [120].

The modification effect can be characterized by various methods. Elemental analysis and thermogravimetry determine the amount of organic modifier incorporated [120]. Fourier-transform infrared spectroscopy (FTIR) and solid-state nuclear magnetic resonance (NMR) provide information on the chemical structure and bonding of the functional groups [121]. N_2_ physical adsorption assesses changes in specific surface area and pore volume after functionalization [122]. X-ray photoelectron spectroscopy (XPS) and scanning electron microscopy (SEM) analyze the surface chemical composition and morphology of the modified SBA-15, respectively [121,122]. These techniques collectively provide insights into the structure–property relationships of functionalized SBA-15 materials.

Similarly, amino-functionalized SBA-15 has been effective in removing cationic dyes like methylene blue and rhodamine B from water, driven by electrostatic interactions [123]. Aminopropyl-functionalized SBA-15 also exhibited outstanding adsorption capacity for humic acid [117] and various VOCs like toluene, ethylbenzene, and xylene [118]. These examples demonstrate the versatility and effectiveness of SBA-15 in addressing pollution challenges in wastewater treatment and environmental remediation.

#### 5.1.2. Adsorption of Heavy Metal Ions

SBA-15 has demonstrated excellent potential in the adsorption of heavy metal ions from aqueous solutions, owing to its tunable physicochemical properties and surface functionalization. Functionalized SBA-15 materials have shown high affinity and selectivity for the adsorption of various heavy metal ions, such as chromium (Cr) [124], copper (Cu) [125], zinc (Zn) [79], cadmium (Cd) [35], and lead (Pb) [126]. The adsorption performance of SBA-15 is influenced by factors such as pH, with optimal removal often observed in the pH range of 4–6. Adsorption studies have revealed that SBA-15 exhibits rapid adsorption kinetics and high adsorption capacities, following both Langmuir and Freundlich isotherm models.

For instance, N-hydroxysuccinimide (NHS)-functionalized SBA-15 materials exhibited excellent adsorption performance for Cu(II) ions, with a maximum adsorption capacity of 138.8 mg/g at pH 5.5. Kinetic studies revealed that the adsorption process followed a pseudo-second-order kinetic model, while the isotherm data fitted well with the Langmuir model, indicating monolayer adsorption. The coordination interaction between NHS groups and Cu(II) ions was proposed as the main adsorption mechanism [123].

Table 4 summarizes the adsorption performance of various functionalized SBA-15 materials for different heavy metal ions.

Furthermore, amine-functionalized SBA-15 materials exhibited high selectivity and adsorption capacity for Zn(II) ions, while the adsorption of Cu(II) and Co(II) ions was relatively lower [124]. This selectivity was mainly attributed to the different coordination strengths between the amine groups and different metal ions. By tuning the type and density of functional groups on SBA-15, selective adsorption of specific metal ions can be achieved.

These findings highlight the significant potential of SBA-15 and its functionalized derivatives in environmental applications, particularly in the treatment of contaminated water. The successful adsorption of organic pollutants and heavy metal ions demonstrates the versatility of these materials in water purification processes. Building upon these achievements in liquid-phase applications, researchers have expanded their investigations to explore the use of SBA-15 in gas adsorption and membrane separation processes. These diverse applications pave the way for SBA-15 materials to play a crucial role in various environmental remediation strategies. The following section will delve into these advanced applications, examining how SBA-15 and its derivatives are being utilized in gas-phase treatments and innovative separation technologies.

### 5.2. Gas Adsorption and Membrane Separation

SBA-15 has been widely explored for the adsorption and separation of gases, such as CO_2_, H_2_, and CH_4_, due to its favorable textural properties and surface chemistry. Surface modification of SBA-15 has been employed to enhance its adsorption capacity and selectivity towards specific gases. The most important factor in post-modifying SBA-15 for gas adsorption applications is the grafting of functional groups onto the pore walls. Amino groups are commonly introduced using silane coupling agents, such as APTES, to functionalize SBA-15 for CO_2_ capture [129]. The yield of this modification process can vary significantly depending on the synthesis conditions, including temperature, pH, and reagent concentrations. Optimizing these parameters is crucial to achieve a high degree of functionalization and improved gas adsorption performance [130].

As discussed in Section 5.1.1, various characterization techniques are employed to validate and quantify the extent of modification in functionalized SBA-15 materials. In the context of gas adsorption applications, these techniques are particularly focused on confirming the successful grafting of amino groups and assessing the impact of modification on the pore structure and surface properties of SBA-15, which are critical factors influencing gas adsorption performance [131].

Amino-functionalized SBA-15 has shown high CO_2_ adsorption capacity and selectivity under simulated gas mixture conditions, making it suitable for post-combustion CO_2_ capture and industrial applications [132]. Studies have demonstrated that shorter pore lengths enhance the adsorption capacity [133,134]. The amino groups on SBA-15 facilitate strong interactions with CO_2_ molecules through chemisorption, making it highly effective for CO_2_ capture from flue gases [132,134].

In addition to its application in gas adsorption, SBA-15 has also been explored as a promising material for the fabrication of high-performance mixed matrix membranes (MMMs) for various separation applications, including hydrogen purification and CO_2_ capture [135]. SBA-15-based MMMs have demonstrated enhanced H_2_ purification and CO_2_ capture performance by leveraging the unique structural features of SBA-15 and the properties of the polymer matrix. The incorporation of SBA-15 into polymer matrices has been shown to significantly improve the gas separation efficiency by overcoming the permeability–selectivity tradeoff [136]. The texture and aging conditions of SBA-15 play a crucial role in determining the gas separation efficiency of the resulting MMMs [137]. Furthermore, functionalization of SBA-15 with deep eutectic solvents (DES) has been explored for the development of mixed matrix polymeric membranes for CO_2_ mitigation, demonstrating their potential for effective CO_2_ mitigation in industrial applications [138].

These examples illustrate the versatility and effectiveness of SBA-15 in gas adsorption and separation applications, highlighting its promise as a material for industrial gas separation processes. The ongoing research in this field continues to explore new functionalization methods to further enhance the performance of SBA-15-based materials in gas separation technologies. In addition to these areas, SBA-15 has also shown potential in membrane separation applications. Expanding beyond gas and liquid separations, researchers have begun investigating the use of SBA-15 for the adsorption and separation of various biomolecules, which will be the focus of the next section.

### 5.3. Applications of Biomolecules

SBA-15 has been investigated for the adsorption and separation of various bio-molecules, such as proteins [139], enzymes [140], and antibodies [141]. The unique structural properties of SBA-15 make it suitable for the adsorption and separation of biomolecules. Surface modification of SBA-15 with organic functional groups or affinity ligands has been employed to enhance its adsorption capacity and selectivity towards specific biomolecules.

Studies have shown that SBA-15 can effectively adsorb biomolecules such as myoglobin, occupying around 50% of the mesopores and following a Langmuir-type monolayer coverage on the inner pore surfaces [140]. The adsorption capacity of biomolecules, such as lysozyme, can be further enhanced by functionalizing SBA-15 with appropriate ligands, such as hyaluronic acid [141]. Moreover, SBA-15 has been widely used for enzyme immobilization, demonstrating improved stability, reusability, and catalytic activity of the immobilized enzymes compared to their free counterparts [142,143].

These studies highlight the potential of SBA-15 as a promising material for biocatalysis, bioseparation, and immunoaffinity applications. The ability to fine-tune the structural and surface properties of SBA-15 through various synthesis and functionalization strategies makes it a versatile platform for the development of advanced biomolecule adsorption and separation systems. Building on these capabilities in biomolecular applications, researchers have expanded the utility of SBA-15 into chromatographic separation processes. The following section will explore how the unique properties of SBA-15 are being leveraged in chromatography, further demonstrating its versatility in separation technologies.

### 5.4. Chromatographic Separation

SBA-15 has been effectively employed as a stationary phase in chromatographic separation applications, such as high-performance liquid chromatography (HPLC) and capillary electrochromatography (CEC) [144,145]. The well-defined pore structure, high surface area, and tunable surface chemistry of SBA-15 contribute to its excellent performance in the separation of a wide range of analytes, including small biomolecules, chiral compounds, peptides, and proteins [144,145,146].

Functionalization of SBA-15 with appropriate moieties, such as C18 groups, cyclodextrins, or nanoparticles, has been shown to enhance its chromatographic performance and selectivity [144,147,148]. For instance, C18-modified mesoporous SBA-15 has been used as a stationary phase in HPLC, resulting in improved separation efficiency, increased resolution, and narrower peak widths compared to conventional C18 columns [144]. Similarly, the incorporation of chiral selectors or nanoparticles onto the SBA-15 surface has led to enhanced enantioseparation and improved separation performance in CEC [147,149].

The versatility and effectiveness of SBA-15 in chromatographic separation applications stem from its unique structural properties and highly tunable surface chemistry. These characteristics make SBA-15 an attractive stationary phase for complex mixtures and challenging analytes, positioning it to play an increasingly important role in the development of advanced separation technologies. As the field of chromatographic separation evolves, SBA-15-based materials are expected to contribute significantly to future innovations. Beyond chromatography, SBA-15 has also emerged as a promising support material for solid–liquid separation processes, further demonstrating its wide-ranging potential in separation technologies. The final section of this chapter will explore these solid–liquid separation applications, providing a comprehensive overview of SBA-15’s versatility across various separation domains.

### 5.5. Solid–Liquid Separation

SBA-15 has emerged as a promising support material for the immobilization of various functional materials, such as enzymes, metal nanoparticles, and polymers, for solid–liquid separation applications. The large surface area, tunable pore size, and rich surface chemistry of SBA-15 make it an ideal platform for the development of efficient and sustainable solid–liquid separation systems.

Immobilization of enzymes on SBA-15 has been shown to improve their stability and reusability, while maintaining high catalytic activity [140,150]. Immobilization of enzymes on SBA-15 has been shown to improve their stability and reusability, while maintaining high catalytic activity [151,152]. Furthermore, the functionalization of SBA-15 with polymers, such as polyethyleneimine (PEI) and chitosan, has led to the development of highly efficient adsorbents for the removal of heavy metal ions and organic pollutants from aqueous solutions [125,153].

The success of SBA-15 in solid–liquid separation applications can be attributed to its unique structural properties and the ability to fine-tune its surface chemistry through various functionalization strategies, as discussed in previous sections. As the demand for efficient and sustainable separation technologies continues to grow, SBA-15-based materials are poised to play a crucial role in addressing the challenges associated with water purification, wastewater treatment, and the recovery of valuable resources from complex mixtures.

## 6. Conclusions and Future Prospects

This review has provided a comprehensive overview of the recent advances in the surface modification of SBA-15 for adsorption and separation applications. The unique structural and physicochemical properties of SBA-15, combined with various surface modification strategies, have led to the development of high-performance adsorbents and separation materials. Specifically, functionalized SBA-15 materials have demonstrated excellent adsorption capacity, selectivity, and stability in a wide range of applications, including the removal of organic pollutants, heavy metal ions, gases, and biomolecules, as well as in chromatographic and solid–liquid separation processes.

Despite the significant progress, several challenges and opportunities for future research have been identified. These include the development of cost-effective and scalable synthesis methods, rational design of SBA-15-based materials with tailored properties, and successful integration into practical applications. Future research efforts should focus on the optimization of synthesis parameters, exploration of novel surface modification strategies, and comprehensive understanding of the structure-performance relationships. Moreover, the long-term stability, regeneration, and reusability of functionalized SBA-15 materials in real-world applications need to be thoroughly investigated. Special attention should be given to overcoming the limitations of SBA-15, such as its high cost and poor mechanical properties, by developing alternative synthesis routes, exploring low-cost precursors, and reinforcing the silica framework with suitable additives.

In conclusion, SBA-15-based materials have shown immense potential in addressing critical environmental and industrial challenges related to adsorption and separation processes. The insights provided in this review are expected to guide future research efforts in developing advanced SBA-15-based adsorbents and separation materials for sustainable applications. By overcoming the identified challenges and seizing the opportunities, researchers can unlock the full potential of these materials, contributing to the development of efficient, eco-friendly, and economically viable solutions for a wide range of separation and purification processes.

## Figures and Tables

**Table 1:** Comparison of key structural and textural properties of SBA-15 with other mesoporous silica materials.

Material	Specific Surface Area (m^2^/g)	Pore Volume (cm^3^/g)	Pore Size (nm)	Structure	Advantages	Ref.
SBA-15	500–1000	0.6–1.2	5–30	Hexagonal	Tunable pore size, thick pore walls, high thermal stability	[42,43]
MCM-41	~1000	High	2–10	Hexagonal	Large surface area, uniform pore size	[44]
MCM-48	1088–1669	1.206–1.263	2–10	Cubic	High surface area, 3D pore structure	[44,45]
MCF	700–1000	0.8–1.2	10–50	Cellular	Large pore volume, interconnected pores	[46]
KIT-6	600–1000	0.8–1.5	5–9	Cubic	Bicontinuous cubic structure, high thermal stability	[47]
HMS	500–1200	0.6–1.0	2–10	Hexagonal	Easy synthesis, tunable pore size	[48]

**Table 2:** Representative metal oxide and non-metal element modified SBA-15: synthesis methods and performance.

Metal Oxide/ Non-Metal Element	Synthesis Methods	Reaction/Application	Performance	Ref.
Al_2_O_3_	One-step wet impregnation	1-butene metathesis to propene	Enhanced conversion and selectivity; stable catalytic activity	[60]
ZnO	Ultrasonic impregnation	Photocatalytic degradation of methylene blue dye	High catalytic activity and stability	[61]
Fe_3_O_4_	Carrier-based synthesis	Activation of persulfate for pollutant removal	Efficient activation; high removal rate of pollutants	[62]
Co_3_O_4_	Not specified	Fischer–Tropsch synthesis	Decreased specific surface area of SBA-15; co-present as Co_3_O_4_	[63]
CeO_2_	Doping	Biodiesel production from Podocarpus falcatus oil	High biodiesel yield under optimized conditions	[64]
Fe_2_O_3_	Wet impregnation	Degradation of methylene blue	70.2% efficiency under visible light irradiation	[65]
Phosphorous acid	Post-grafting	Adsorption of Gd(III)	Excellent adsorption capacity and kinetics; good reusability	[66]

**Table 3:** Preparation method and performance of inorganic–organic composite SBA-15 materials.

Ionic Liquid	Catalyst	Preparation Method	Reaction	Yield	Ref.
Pyridinium	SBA-15-Py-SO3H	-	Esterification	88% (after 5 cycles)	[89]
Imidazolium	Pd/SBA-15-Im	-	Suzuki coupling	95%	[93]
Phosphonium	SBA-15-P(C6H13)3Br	-	Knoevenagel condensation	92%	[96]
Brønsted-Lewis	Zr-SBA-15/[mim-ps] Cl-ZnCl2	Wet impregnation	Esterification of acetic acid with BnOH	93.6%	[97]
NMImBr	NMImBr-SBA-15	-	Propylene oxide and CO_2_ to propylene carbonate	98.23%	[98]
Alkyl-functionalized imidazolium	BCL/IL-SBA-15	Modification of SBA-15 with alkyl-functionalized ionic liquids	Hydrolysis of triacetin	2.4 times higher than BCL/SBA-15	[99]
Propyl-SO3H	Propyl-SO3H-SBA-15	Microwave-mediated synthesis	Synthesis of multi-substituted imidazoles	-	[100]

**Table 4:** Adsorption performance of functionalized SBA-15 materials for heavy metal ions.

Functionalized Material	Heavy Metal Ion(s)	Maximum Adsorption Capacity (mg/g)	Optimal pH	Isotherm Model	Ref.
Magnetic SBA-15 nanosorbent	Cd(II)	140	5	-	[35]
Crown-ether-modified SBA-15	Zn(II)	-	5	Langmuir	[79]
Amine-functionalized SBA-15	Cu(II), Cr(III), Co(II), Ni(II), Cd(II), Mn(II), Na(I)	-	5–6	-	[125]
Urea-functionalized SBA-15	Cr(VI)/Cd(II)/Pb(II)	26.83/30.53/43.85	2.5/5/4	Langmuir	[126]
Imidazole-derivatized SBA-15	Cr(VI)	113	4–5.5	-	[127]
N-hydroxysuccini mide-functionalized SBA-15	Cu(II)	138.8	5.5	Langmuir	[128]

## Data Availability

No new data were created or analyzed in this study. Data sharing is not applicable to this article.

## Abbreviations

2-HB	2-Hydroxybenzaldehyde
4-HB	4-Hydroxybenzaldehyde
AO	Amidoxime
APTES	3-Aminopropyltriethoxysilane
BHET	Bis(2-Hydroxyethyl) Terephthalate
CEC	Capillary Electrochromatography
CMK-3	Carbon Mesostructured by KAIST-3
CMK-5	Carbon Mesostructured by KAIST-5
DES	Deep Eutectic Solvents
DM	Direct Modification
Dox	Doxorubicin
Fe_3_O_4_@SBA-15	Fe_3_O_4_-wrapped mesoporous molecular sieve catalyst
Fe_3_O_4_@SBA-15-Gd	Magnetic Fe_3_O_4_@SBA-15-Gd nano-adsorbent
Fe_3_O_4_@SBA-15-NH_2_	Fe_3_O_4_@SBA-15 modified with 3-aminopropyltriethoxysilane
FTIR	Fourier-Transform Infrared Spectroscopy
Ga-SBA-15	Gallium-substituted SBA-15
HPLC	High-Performance Liquid Chromatography
IL	Ionic Liquid
La-IIP/SBA-15/Y	Lanthanum ion-imprinted polymer on composite molecular sieve SBA-15/Y
MCM-41	Mobil Composition of Matter No. 41
MCM-48	Mobil Composition of Matter No. 48
Melamine-MS-SBA-15	Melamine functionalized mesoporous silica-SBA-15
MMMs	Mixed Matrix Membranes
MOFs	Metal-Organic Frameworks
NH_2_-H-SBA-15	NH_2_-functionalized hydroxylated mesoporous SBA-15
NMR	Nuclear Magnetic Resonance
P123	Pluronic P123
P-SBA-15	Phosphorous Acid Modified SBA-15
PAA	Phosphonoacetic Acid
PAN	Polyacrylonitrile
PAO	Poly amidoxime
PAO/AO-SBA-15	Amidoxime-functionalized PAN/AO-SBA-15 membrane
PEI	Polyethyleneimine
PET	Polyethylene Terephthalate
PFO	Palm Fatty Oil
PS	Persulfate
SBA-15	Santa Barbara Amorphous-15
SBA-15@AMI NPs	Amidoxime functionalized mesoporous silica nanoparticles
SDAs	Structure-Directing Agents
SEM	Scanning Electron Microscopy
SV	Sodium valproate
TEM	Transmission Electron Microscopy
TEOS	Tetraethyl Orthosilicates
VOCs	Volatile Organic Compounds
XPS	X-ray Photoelectron Spectroscopy
XRD	X-ray Diffraction
